# Looking beyond endotoxin: a comparative study of pyrogen retention by ultrafilters used for the preparation of sterile dialyis fluid

**DOI:** 10.1038/srep06390

**Published:** 2014-09-17

**Authors:** Griet Glorieux, Michael Hulko, Rose Speidel, Katrin Brodbeck, Bernd Krause, Raymond Vanholder

**Affiliations:** 1 Nephrology Division, University Hospital Gent, 9000 Gent, Belgium; 2 Gambro Dialysatoren GmbH, Research & Development, 72379 Hechingen, Germany

## Abstract

Sterile single-use ultrafilters are used in dialysis for the preparation of the substitution fluid given to patients undergoing dialysis treatments with high convective fluid removal. The retention of pyrogenic agents by the ultrafilters is crucial to avoiding inflammatory responses. The performance of a new single-use ultrafilter (NUF) with a positively charged flat sheet membrane of relatively small membrane area and large pore size was compared to a reference ultrafilter (RUF) with a hollow fiber membrane. Filter performance was tested with various pyrogen-contaminated dialysis fluids by direct pyrogen quantification and by measuring inflammatory responses in cell-based bioassays. The NUF completely retained oligodeoxynucleotides (ODN), whereas the RUF was fully permeable. Both filters tended to decrease biological activity of DNA in filtered bacterial lysates. The NUF reduced lipopolysaccharides (LPS) and LPS-induced biological activity by 100%, whereas the RUF produced filtrates with low but detectable levels of LPS in most cases. Peptidoglycans (PGN) were fully retained both by the NUF and the RUF. The new ultrafilter retained biologically active ODN, which has not yet been described for any other device used in dialysis, and it showed better or equal retention of LPS and PGN even with a smaller membrane surface and larger pore size.

High flux membranes for dialysis were introduced to reduce the morbidity and mortality of hemodialysis patients by offering better removal than low flux membranes of large uremic retention solutes. Large uremic solute removal with high flux membranes can be further improved in a hemodiafiltration (HDF) setting. This setting allows high fluid flows and convective transport, providing the most effective removal of the widest range of uremic retention solutes. Three controlled trials compared HDF therapy with low-flux hemodialysis (CONTRAST^1^), with high-flux hemodialysis (TURKISH^2^) and with hemodialysis (ESHOL^3^) These studies suggest a possible survival advantage only when high convection volumes can be delivered (> 20 l).

In an HDF setting, fluid removed from the patient needs to be substituted, and the quality of substitution fluid is of utmost importance, particularly for those patients treated with high exchange volumes. Modern dialysis machines prepare substitution fluid on-line, meaning that the fluid is continuously mixed by an on-line preparation system consisting of water, concentrate supply and ultrafilters. Fluid prepared on-line is given to the patient immediately after its preparation (Figure 1). The requirements for on-line preparation of sterile and non-pyrogenic substitution fluid are described in the ISO11663 standard^4^.

Contamination of dialysis water and fluid by water-borne microorganisms gives rise to pyrogenic fragments of microorganisms, which are a special matter of concern. Among the fragments are lipopolysaccharides, peptidoglycans, and short fragments of bacterial deoxynucleic acid (DNA). Each of these substances can be detected by substance-specific assays or by bioassays with broader selectivity, and each of these substances is known for their potential to cause an inflammatory response after transfer into the blood^5^.

On-line fluid preparation systems typically comprise a cascade of multiple ultrafilters to maintain the required fluid quality^6^. One possible ultrafilter configuration includes a first ultrafilter installed within the dialysis machine that purifies the incoming pretreated water; next, in the machine, this ultrafiltered water is mixed with acid and bicarbonate concentrate delivered by a cartridge to generate dialysis fluid which is subsequently filtered through a second ultrafilter installed in the dialysis machine. These machine-installed ultrafilters are disinfected after each treatment and remain in the machines for a defined number of recurrent uses. At this point, ultrapure dialysis fluid containing less than 0.1 colony forming units per ml and less than 0.03 endotoxin units (EU)/ml can be achieved^4^. Sterile substitution fluid for infusion (Sterility Assurance Level (SAL) 6 and < 0.03 EU/ml) can be generated in a third filtration step by filtering ultrapure dialysis fluid through a sterile single-use ultrafilter.

In the present study, a new sterile single-use ultrafilter was benchmarked against a reference ultrafilter with respect to retention of lipopolysaccharides, peptidoglycans and short bacterial DNA fragments. For this purpose, target amounts of the relevant contaminants, based on the highest reported concentrations in dialysis fluid^7^,^8^, were applied to represent the total contamination load in a maximum volume of 50 l of dialysis fluid to be filtered for the preparation of substitution fluid for one dialysis session. These levels of contamination allowed the distinction between full retention and (even) limited leakage.

## Results

### Pore sizes of ultrafilters

The average pore size of the reference ultrafilter (RUF) was estimated to be 4.2 ± 0.1 nm and therefore, approximately 50 times smaller than the nominal average pore size of 0.2 µm of the new UF (NUF).

### Retention of oligodeoxynucleotides

The challenge solution containing a 19mer CG-rich oligodeoxynucleotide (ODN) (1000 ng/ml, 0.17 µM), and the corresponding filtrates of the NUF and the RUF were quantitatively analyzed by the Quant-iT OliGreen Assay. The NUF reduced ODN in the challenge solution containing 930 ± 42 ng ODN/ml to levels below the detection limit of 2 ng/ml, corresponding to the background level of the buffer. In contrast, the RUF did not retain ODN (filtrate, 841 ± 44 ng/ml vs. challenge: 854 ± 31 ng/ml) (Figure 2).

These data were confirmed by demonstrating the change of biological activity after filtration of the ODN-containing challenge solution by the reduction of interleukin-1-beta (IL-1β) mRNA expression in peripheral blood mononuclear cells (PBMC) measured by quantitative reverse transcription polymerase chain reactions (qRT-PCR). The challenge solution in the experiment with the NUF induced a 1.47 ± 0.24-fold IL-1β mRNA expression relative to buffer, whereas after filtration, the IL-1β mRNA expression dropped to 1.16 ± 0.31-fold relative to buffer. In contrast, the challenge solution of the RUF induced 1.27 ± 0.25-fold IL-1β mRNA expression relative to buffer, and the IL-1β mRNA expression induced by the filtrate of the RUF was 1.37 ± 0.35-fold (Figure 3). All these data indicate the full retention of bacterial oligonucleotides by the NUF, whereas the RUF was completely permeable.

### Retention of lipopolysaccharide

Ultrafilters were tested for their retention of lipopolysaccharide (LPS). A challenge solution containing LPS from the gram-negative bacterium, *Pseudomonas aeruginosa*, with a response in the limulus amebocyte lysate (LAL) test of 436 ± 132 endotoxin units (EU)/ml was prepared. After filtration with the NUF, the response in the LAL test of the filtrate dropped below the detection limit of < 0.005 EU/ml. The filtrate of the RUF still contained 6.26 ± 0.94 EU/ml (1.99 ± 0.37% was not retained) (Figure 4).

These results were confirmed by the change in biological activity after filtration of the challenge solution contaminated with LPS, illustrated by the reduction or absence of cytokine (IL-1β) inducing capacity (CIC) of the filtrates in the THP-1 cytokine induction assay (CIA). In the filtrates obtained with the NUF, no CIC was detectable, while 18.79 ± 4.33% of the CIC remained in the RUF filtrates (Figure 5).

The data from these experiments show that the NUF offers a better retention performance for LPS than the RUF.

### Retention of peptidoglycan

No peptidoglycan (PGN) could be found with the silkworm larvae plasma (SLP) test in the filtrates of either the NUF or the RUF. However, these quantitative SLP tests were not reliable because an inhibition of the reaction was observed with the “sample control” (buffer spiked with PGN) (< 1% of the spike was retrieved), and the detected concentrations of the challenge solution (6.1 ± 2.6 ng/ml) were below 1% of the expected spiked concentration (1000 ng/ml). For these reasons, the quantitative analysis of PGN was omitted, and only the results of the bioassays [THP-1 and pattern of recognition receptors (PRR)] were considered in evaluating PGN retention. The THP-1 CIA revealed that after filtration of the challenge solution contaminated with *Bacillus subtilis* PGN (1000 ng/ml PGN resulted in 103.5 ± 20.73 pg/ml of IL-1β), the CIC of the NUF filtrates dropped to 11.23 ± 1.18 pg/ml of IL-1β, and the CIC of the RUF filtrates dropped to 11.30 ± 2.09 pg/ml of IL-1β. These values were comparable to the baseline levels (13.72 ± 5.96 pg/ml IL-1β). In addition to *B. subtilis* PGN, *Staphylococcus aureus* PGN (1000 ng/ml) was tested and resulted in a response of >307 pg/ml IL-1β (out of range) in the challenge solution. Again, no CIC was observed in the filtrates of either the NUF or the RUF (Figure 6).

For confirmation of the findings of the THP-1 CIA, the challenge solution with *S. aureus* PGN and the corresponding filtrates of the NUF and the RUF were further analyzed with PRR bioassays. As illustrated in Figure 7, the PGN challenge solution activated the cells as reflected in the increased absorbance values. Activation via the toll-like receptors TLR1/2, TLR2/6, TLR4/CD14 (selective for lipopolysaccharides) and TLR9 (selective for bacterial DNA) was observed, pointing to the fact that the commercially obtained *S. aureus* PGN was most likely not completely pure and contained traces of LPS and bacterial DNA. However, this contaminated PGN solution gave no detectable response in the LAL test.

The filtrate solutions obtained with the NUF and the RUF showed reduced activation, down to background level, of all TLR-receptors (Figure 7). These data confirm the complete retentive capacity for biologically active PGN by both ultrafilters, consistent with the THP-1 CIA described above.

### Retention of bacterial lysates from *P. aeruginosa*

The response to a challenge solution containing 12.5 × 10^6^ colony forming units (CFU)/l of *P. aeruginosa* and to its filtrates was analyzed by the LAL test, the Quant-iT OliGreen assay and the THP-1 CIA and PRR bioassays. The challenge solution induced a response in the LAL test of 6.29 ± 1.11 EU/ml, which dropped below the detection limit (< 0.005 EU/ml) after filtration with the NUF, whereas after filtration with the RUF, the LPS levels, 0.039 ± 0.012 EU/ml (0.60 ± 0.12% not retained), remained above the detection limit. ODN could not be detected in the challenge solutions or the filtrates. The challenge solutions containing gram-negative *P. aeruginosa* lysates, and the corresponding filtrates of the NUF and the RUF were further analyzed in the THP-1 CIA. The CIC of the challenge solution was 211 ± 43 pg/ml of IL-1β, which dropped to baseline levels (8.58 ± 0.50 pg/ml IL-1β) after filtration with either the NUF (12.20 ± 2.81 pg/ml IL-1β) or the RUF (9.67 ± 0.77 pg/ml IL-1β). Analysis with the PRR assay demonstrated a significant and strong activation of TLR4/CD14 (selective for lipopolysaccharides, p < 0.05 vs buffer). In addition, there was activation of TLR1/2 and TLR2/6, selective for peptidoglycans and other cell-wall fragments, and a trend toward activation of TLR9, selective for DNA. The filtrates of both the NUF and the RUF showed reduced activation of all TLR-receptors down to background level, meaning reductions of 100% (Figure 8).

## Discussion

Prototypes of a new single-use flat sheet membrane ultrafilter were benchmarked against a reference ultrafilter with regard to the retention of known bacteria-derived pyrogens. In the past, systems for the preparation of dialysis fluid were only tested for the retention of intact microorganisms and intact LPS according to the ISO norm^4^. However, other pro-inflammatory bacterial derivatives, including PGN and bacterial DNA, definitely contaminate dialysis fluids^7^,^8^. Thus, the evaluation methods applied in the present study were extended beyond the classical definitions to encompass all contaminants with inflammatory potential.

One of the most interesting findings of this study was the lack of retention of ODNs by the reference ultrafilter compared to the complete retention by the new ultrafilter. The presence of bacterial ODN in dialysate samples has been reported by Schindler et al., and concentrations of 0.3 µg/ml were detected. Those ODNs were shown to have inflammatory capacity^7^. The latter observation was confirmed by showing that bacterial DNA enhances cytokine production and promotes the survival of inflammatory mononuclear cells from chronic kidney disease patients^9^. More specifically, the inflammatory response induced by the stimulation of the CD14^+^CD16^+^ pro-inflammatory monocytic subpopulation^10^,^11^ by CpG DNA was shown to result in endothelial cell apoptosis^11^. In addition, Bossola et al. identified bacterial DNA in the blood of hemodialysis patients and found that circulating bacterial DNA is associated with higher levels of C-reactive protein and interleukin-6 in hemodialysis patients^12^. Thus, especially when infusing purified dialysis fluid, prevention of the transfer of ODNs into the blood should be a primary aim to avoid pro-inflammatory effects contributing to cardiovascular disease in hemodialysis patients.

The direct quantification of ODN revealed the clearest difference between the NUF that reduced the ODN content of the challenge solution down to undetectable levels in the filtrates and the RUF that reduced ODN content only slightly. Beyond direct ODN quantitation, bacterial DNA retention was also studied indirectly with the PRR bioassay via the TLR9 response. The NUF retained ODN and bacterial DNA in all tested cases, while the RUF was completely permeable to ODN as revealed by the quantitative data (Figure 2). But, the ODN permeability of the RUF could not be inferred from the results of the PRR bioassays. It is of note that, to obtain a detectable response, a minimal concentration of 5 µg/ml ODN is needed for TLR9 in the PRR test system^13^, meaning that the quantitative OliGreen assay is the more sensitive test and that ODN concentration below this value might remain undetected by the PRR test system. Therefore, the biological activity of ODN was assessed with a test system that was expected to be more sensitive than the PRR bioassay, namely qRT-PCR analysis of IL-1β mRNA expression in PBMC. The results of qRT-PCR analyses were in alignment with the quantitative ODN measurements, which showed that the NUF reduced ODN-induced cell activity down to baseline levels, whereas the RUF had no effect.

Three retention mechanisms have been described in the literature: size exclusion^14^, hydrophobic adsorption^15^, and electrostatic adsorption^16^. According to the latter retention mechanism, the positively charged membrane of the NUF should be particularly efficient in retaining negatively charged bacterial contaminants, including LPS, PGN and bacterial DNA, in bicarbonate dialysis fluid. The retention mechanism of ODN by the NUF is most likely based on an electrostatic charge interaction between the negatively charged ODN molecules and the positively charged membrane. Retention by size exclusion of ODN is unlikely because the nominal pore size of the NUF is larger than the molecular dimensions of the tested ODN. The electrostatic charge density of oligonucleotides is determined by the phosphate-deoxyribose backbone of the ODN molecule, and therefore, it can be assumed that the retention characteristics do not depend on a specific sequence of the nucleobases. The capacity to completely retain biologically active ODN as observed with the NUF has, to the best of our knowledge, not yet been described for any other device applied in dialysis.

The new ultrafilter, NUF, also retained LPS more efficiently than did the reference ultrafilter. No detectable amount of LPS could pass the NUF in contrast to the RUF. Dialysis machines are able to generate ultrapure dialysis fluid with LPS levels lower than 0.03 EU/ml^4^ that passes the single-use ultrafilter to generate sterile substitution fluid. Thus, additional LPS retentive capacity of the single-use final ultrafilter might not be of relevance as long as the fluid systems are properly operated and maintained. Nevertheless, this retentive capacity might be very useful even with minor contamination, especially because the latter is rarely detected immediately, even with correct and regular monitoring of dialysis water and fluid quality. Moreover, controls of water quality at regular intervals as recommended^17^ might often be neglected. This risk becomes, by definition, greater as the retentive capacity of a given ultrafilter becomes lower. It might seem counterintuitive that the NUF with a larger nominal pore size and a smaller membrane area retains LPS better than other ultrafilters with larger membrane areas and smaller pore sizes, but retention based on charge is very likely the principal mechanism at play in this context. In view of the high contamination levels used in the present experimental set-up, mimicking an LPS contamination of 10 EU/ml in the dialysis fluid (ISO 11663:2009: < 0.5 EU/ml), which is highly unlikely in reality, saturation of the new single use ultrafilter is not to be expected during the course of a single dialysis session.

Biologically active peptidoglycans were equally and completely retained by the NUF as well as by the RUF. PGN retention was demonstrated by assessing the biological activity of PGN by the THP-1 CIA and the PRR bioassay. Remarkably, the PRR bioassay also suggested a co-contamination of biologically active LPS and bacterial DNA in the *S. aureus* PGN solution, contaminants which were no longer traceable in the filtrates of either the NUF or the RUF.

To assess the retention properties under conditions that reflect a more complex mixture of pyrogens, lysates *of P. aeruginosa* were tested. Here also, for both the evaluated ultrafilters (NUF and RUF), no difference in the LPS and PGN retention capabilities was observed. It is, however, of note that the degree of contamination in the challenge solutions based on bacterial lysates was substantially lower than in the test solutions containing purified contaminants.

Beyond the benchmarking aspect, our study raises the question whether current recommendations for checking dialysis water and fluid purity^18^ are sufficient to detect all important potential contaminants. This point is particularly relevant in case of on-line hemodiafiltration compared with conventional hemodialysis because potential contaminants can enter the blood stream not only by diffusion but also by active infusion. One might object that the spiking experiments, as performed here, do not conform to real-life circumstances. Pyrogenic substances used to challenge the ultrafilters were selected according to three criteria: the relevance of the nature of the contaminants, their concentrations in a dialysis setting and the availability of methods for quantification and evaluation of biological activity^5^. Even more striking, for some ultrafilters and some substances, including the ODNs, virtually no removal was observed with the currently used ultrafilters. Therefore, the discussion about water purity and water purity standards is still relevant. Finally, the new ultrafilter performs better with respect to the retention of LPS and bacterial DNA, the latter being a novel finding that has not been described for any other device.

## Methods

### Fluids

Dialysis fluid (“buffer”) was prepared with water purified by reverse osmosis, acid concentrate (D242; 2–4 mmol/l) and bicarbonate concentrate (D200; 30–40 mmol/l) to a final pH of 7.1–7.5 (Gambro Hospal, Kilchberg, Switzerland).

### Ultrafilters

In the benchmark, prototype devices of a new single-use ultrafilter (provided by Gambro Dialysatoren, Hechingen, Germany) were used. The NUF was beta-sterilized and contained a flat-sheet membrane made of polyethersulfone with a membrane area of 25 cm^2^, a thickness between 114 and 140 µm and a nominal pore size of 0.2 µm. The membrane was modified so that a positive charge was created by adding quaternary ammonium groups to the membrane surface. The housing was made of polyethylene terephthalate glycol. For comparison, a commercially available single-use ultrafilter (provided by Gambro Dialysatoren, Hechingen, Germany) intended for ultrafiltration of fluids was used as the reference. The RUF was gamma-sterilized and contained hollow fiber membranes based on polyethersulfone/polyvinylpyrrolidone blends with inner diameters of approximately 200 µm, a membrane thickness between 35 and 50 µm and a membrane area of 2000 cm^2^. The pore size of the RUF was estimated by means of the Einstein-Stokes radius by the filtration of dextran mixtures^19^. The housing was made of polycarbonate and polyurethane was used for fiber potting.

### Challenge solutions

The following purified contaminants were used: lipopolysaccharide (500 EU/ml) from *P. aeruginosa* (Sigma, St. Louis, MO, USA), peptidoglycan (1000 ng/ml) from *B. subtilis* (InvivoGen, San Diego, CA, USA), PGN from *S. aureus* (InvivoGen, San Diego, CA, USA) and oligodeoxynucleotides (1 µg/ml, 19mer, 5′ TCGACTCTCGAGCGTTCTT) according to the sequences published by Schindler et al.^7^,^8^ with a molecular weight of 5750 Da (TIB Molbiol, Berlin, Germany). The ODN solution used for bioassays was filtered with the RUF immediately after preparation, minimizing the risk of inducing a response to potential contaminating pyrogens other than ODN.

Contaminant amounts in challenge solutions were chosen based on the highest reported concentrations detected in dialysis fluid^7^,^8^ and were adjusted to represent the total contamination load in a maximum volume of 50 l of dialysis fluid to be filtered by ultrafilters for the preparation of substitution fluid during one dialysis session. When meeting the ISO 11663:2009 standard^4^ for LPS allowing < 0.5 EU/ml in dialysis fluid, a maximum LPS load in one dialysis session (50 l) equals 25,000 EU. To mimic this in our experimental setting, filtering only 1 l, the dialysis fluid must contain at least 25 EU/ml.

To create a more complex composition of contaminants, lysates directly derived from bacterial cultures of *P. aeruginosa* (Gram negative, a major source of LPS; BCCM/LMG, Gent, Belgium) were used as well. The cultures with a final concentration of 12.5 × 10^6^ CFU/l of *P. aeruginosa* were harvested, treated with heat (20 min at 95°C) and ultrasound (1 min at 10 rpm) to induce bacterial disintegration and then added to the dialysis fluid (buffer). For each filtration experiment, fresh solutions were prepared.

### Filtration set-up

**Figure 1:**
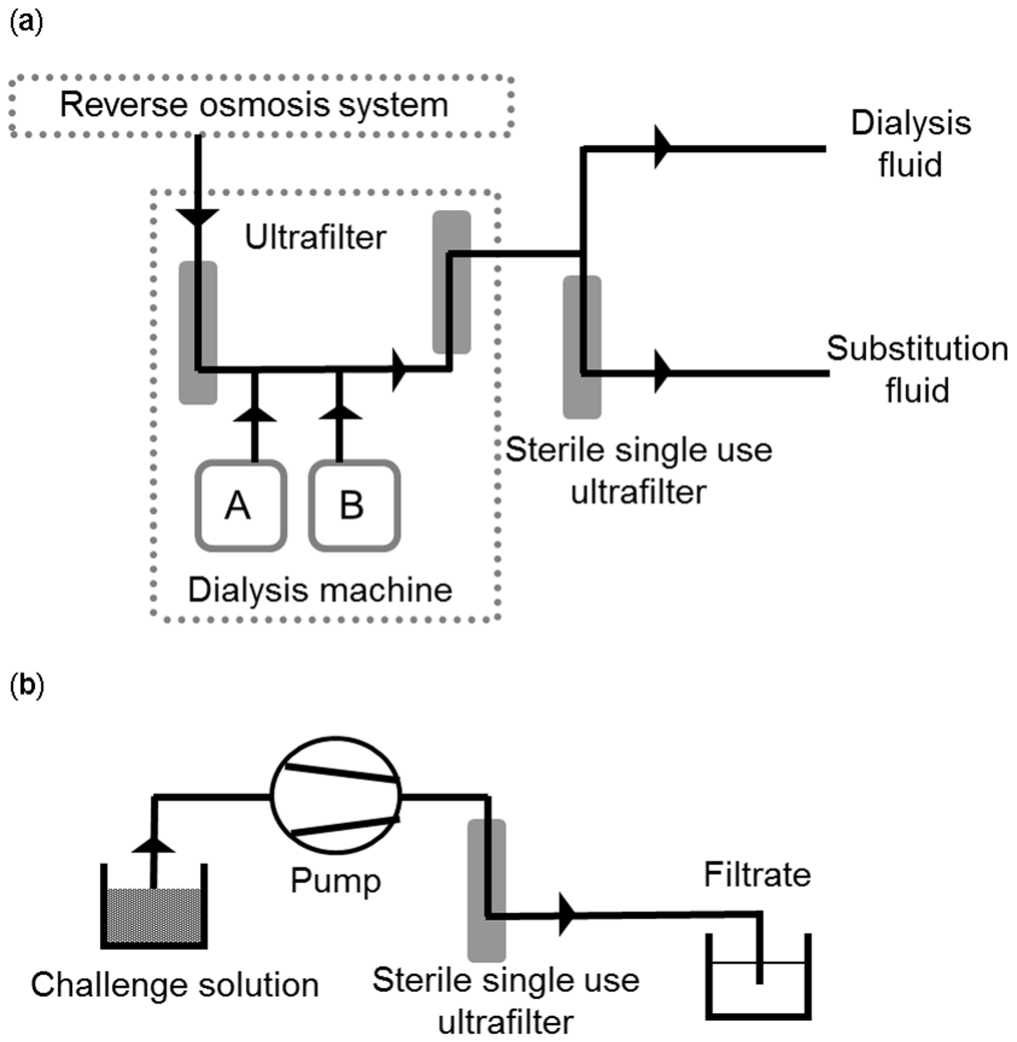
Flow charts of on-line dialysis fluid preparation. (a) The three ultrafilters concept: reverse osmosis water is purified by a first ultrafilter. In the machine, water is mixed with acid (A) and bicarbonate concentrate (B) and processed through a second ultrafilter, resulting in ultrapure dialysis fluid. Sterile substitution fluid for infusion is generated by a third filtration step with a sterile single use ultrafilter. (b) The filtration set-up for the benchmark study. The challenge solution is pumped across the membrane to obtain the filtrate in a single-pass filtration step.

**Figure 2:**
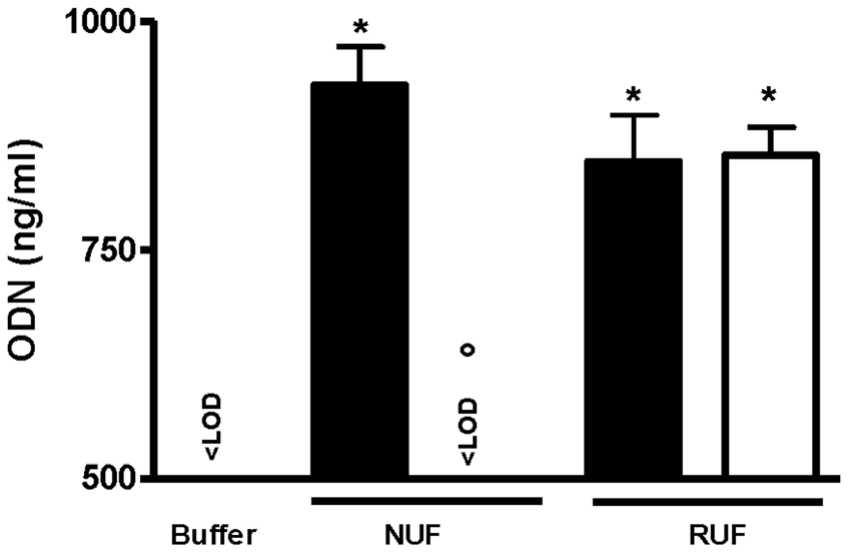
Retention of ODN, assessed by measuring ODN concentrations (ng/ml) by Quant-iT OliGreen assays. New ultrafilter (NUF); Reference ultrafilter (RUF); Challenge solution (black bars); Filtrates (white bar), LOD = 2 ng/ml; *P < 0.001 versus buffer and °P < 0.001 versus the corresponding challenge solution; n = 3.

**Figure 3:**
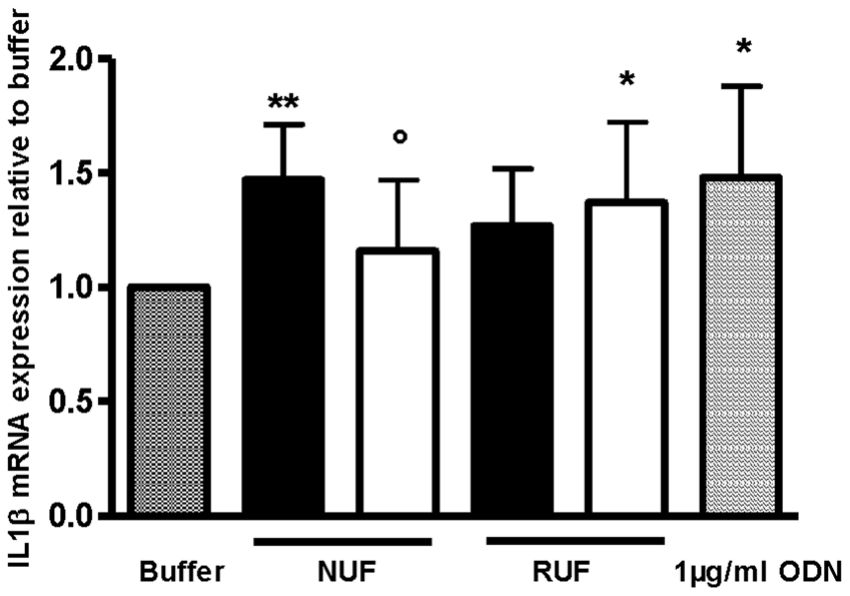
Retention of ODN assessed by measuring IL-1β mRNA expression levels induced in PBMCs (cytokine induction assay). The IL-1β mRNA expression levels were measured by qRT-PCR. New ultrafilter (NUF); Reference ultrafilter (RUF); Challenge solutions (black bars); Filtrate (white bars); **P < 0.001; *P < 0.01 versus buffer and °P < 0.05 versus corresponding challenge solution; n = 3.

**Figure 4:**
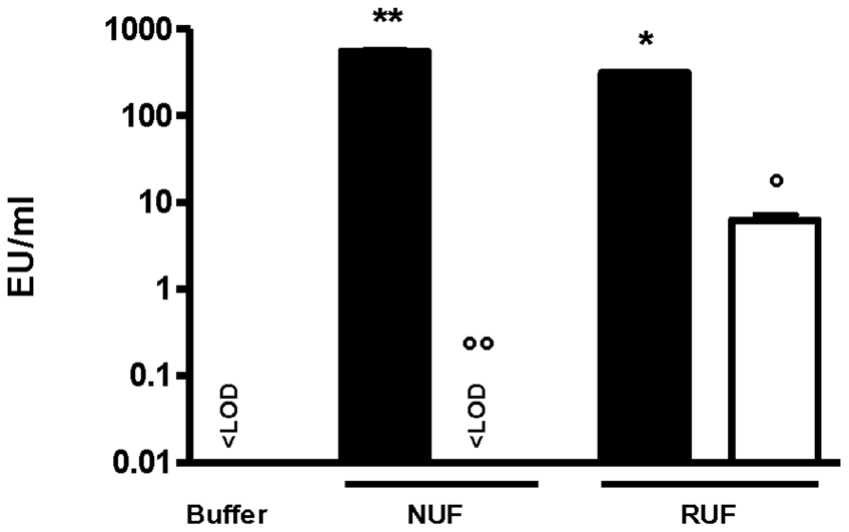
Retention of *P. aeruginosa* LPS assessed by determining LPS concentrations (EU/ml) with the LAL test. New ultrafilter (NUF); Reference ultrafilter (RUF); Challenge solutions (black bars); Filtrates (white bar); LOD = 0.005 EU/ml; **P < 0.001; *P < 0.01 versus buffer and °°P < 0.001; °P < 0.01 versus corresponding challenge solution; n = 3.

**Figure 5:**
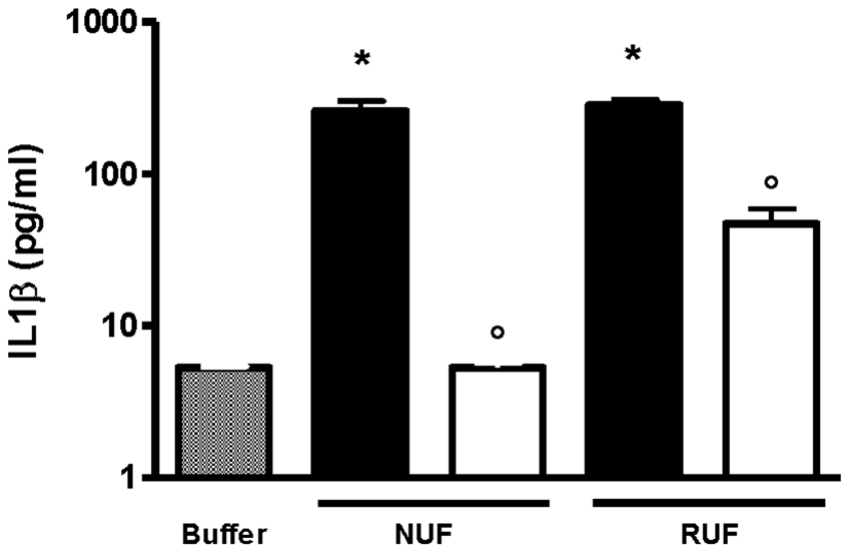
Retention of *P. aeruginosa* LPS assessed by estimating cytokine (IL-1β pg/ml) induction capacity (CIC) in THP-1 cell cultures. The IL-1β was measured in the supernatant by ELISA. New ultrafilter (NUF); Reference ultrafilter (RUF); Challenge solution (black bars); Filtrates (white bars); *P < 0.001 versus buffer and °P < 0.001 versus corresponding challenge solution; n = 3.

**Figure 6:**
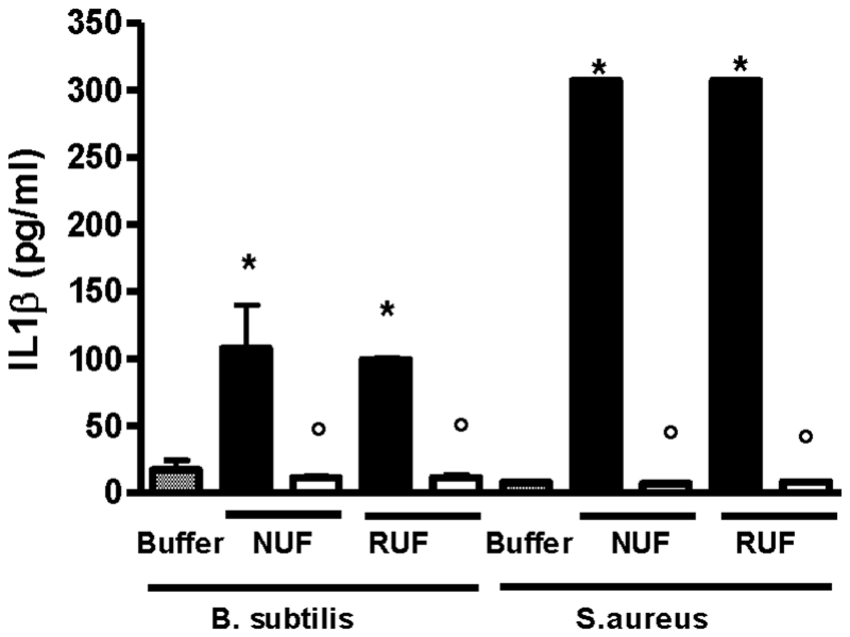
Retention of PGN [*B. subtilis* and *S. aureus*] assessed by estimating cytokine (IL-1β pg/ml) induction capacity (CIC) in THP-1 cell cultures. The IL-1β was measured in the supernatant by ELISA. New ultrafilter (NUF); Reference ultrafilter (RUF); Challenge solution (black bars); Filtrates (white bars); *P < 0.001 versus buffer and °P < 0.001 versus corresponding challenge solution; n = 3.

**Figure 7:**
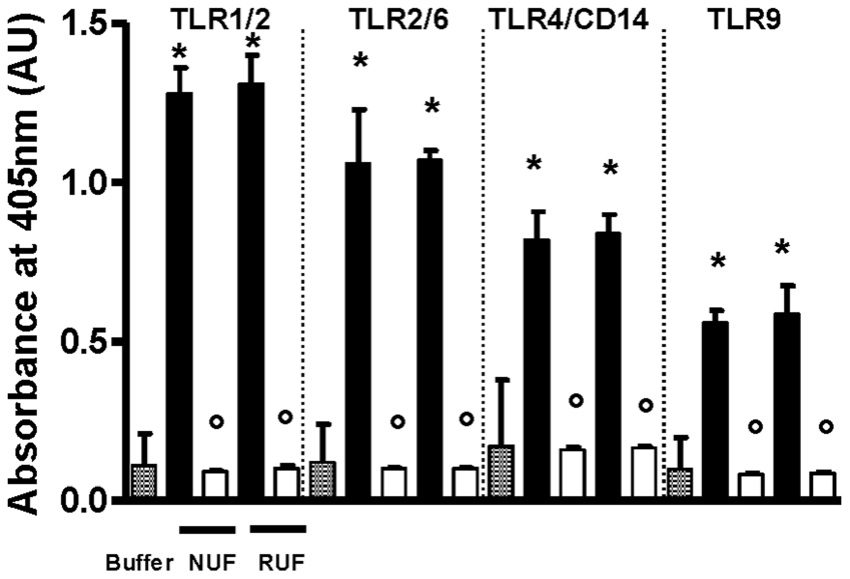
Retention of PGN (*S. aureus*) estimated by the pattern of recognition receptors (PRR) bioassay (405 nm). New ultrafilter (NUF); Reference ultrafilter (RUF); Challenge solution (black bars); Filtrates (white bars); *P < 0.001 versus buffer and °P < 0.001 versus corresponding challenge solution; n = 3.

**Figure 8:**
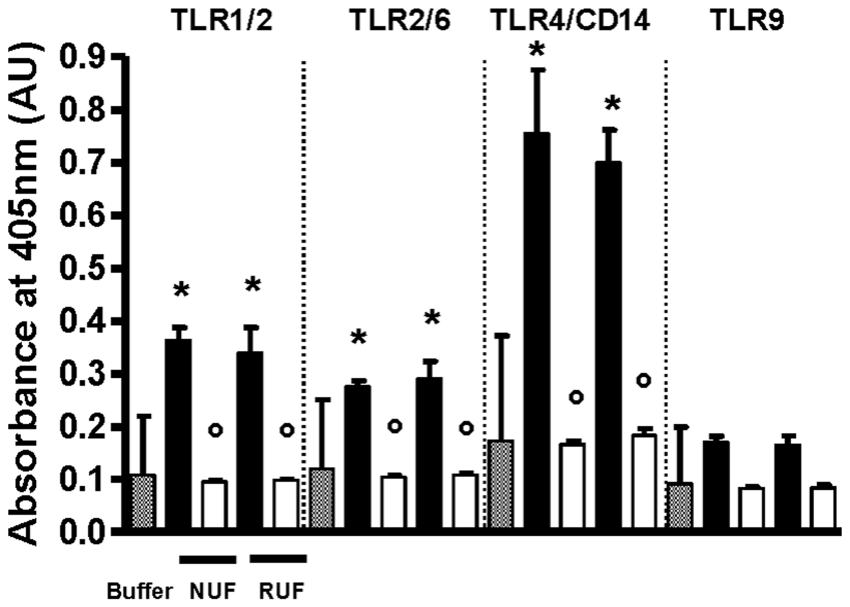
Retention of bacterial lysates (*P. aeruginosa*) estimated by the pattern of recognition receptor (PRR) bioassay (405 nm). New ultrafilter (NUF); Reference ultrafilter (RUF); Challenge solution (black bars); Filtrates (white bars); *P < 0.001 versus buffer and °P < 0.001 versus corresponding challenge solution; n = 3.

The filtration for the benchmark was a single-pass filtration in alignment with the respective clinical mode of application (Figure 1). In the case of the hollow-fiber membrane of the RUF, the filtration direction was from the lumen to the outside. The filtration procedure comprised additional steps of priming the filters with buffer to remove air and displacing the priming solution with the challenge solution prior to the final single-pass filtration of 1 l challenge solution. A roller pump was used to maintain a flow rate of 200 ml/min. Pyrogen concentrations were measured in the initial challenge solution and in the total unfractionated filtrates. Pyrogen concentrations were measured in the priming solution as a control to exclude any contamination of the experimental set-up itself.

Three independent experimental repeats were conducted for the NUF and the RUF and for each type of test solution.

### Test systems for quantification

The Quant-iT OliGreen Assay (Invitrogen, NY, USA), which is based on fluorescence enhancement by dye intercalation, was used for the quantification of oligodeoxynucleotides. The assay was performed according to manufacturer's instructions, and fluorescence was determined using a FLUOstar OPTIMA fluorescent plate reader with 480 nm excitation and 520 nm emission wavelengths (BMG Labortech GmbH, Ortenberg, Germany). The detection limit was 2 ng/ml.

The Limulus Amebocyte Lysate test, a kinetic chromogenic assay (Kinetic-QCL R®) (Lonza, Walkersville, MD, USA) with a detection limit of 0.005 EU/ml, was used for quantification of intact LPS according to the manufacturer's guidelines. Escherichia coli 055:B5 was used to establish a standard curve.

The silkworm larvae plasma test (Wako Pure Chemical Industries, Osaka, Japan) was used according to the manufacturer's guidelines to estimate the concentration of peptidoglycan. The limit of detection was 1 ng/ml.

### THP-1 cytokine induction assay

The human monocytic cell line THP-1 (ATCC, LGC Promochem, Middlesex, UK) was maintained as a continuous culture. Cell cultures (1 × 10^6^ cells/ml) were treated for 72 h with calcitriol (10 nM) (a gift from Roche Pharmaceuticals, Basle, Switzerland) after which the medium was refreshed followed by a 24 h rest period before the cells were used. The differentiated THP-1 cells were incubated in a 1:1 dilution (with a total volume of 700 μl) with the test solutions (dialysis fluid or priming solution filtrate) in polystyrene, pyrogen-free culture plates (Nunc, Roskilde, Denmark) for 24 h in a humidified atmosphere of 5% CO2 in air at 37°C. After incubation, the culture suspensions were collected and stored at −20°C. After a centrifugation step (5 min at 3000 rpm) IL-1β expression was quantified in the culture supernatant using a sandwich ELISA kit (Quantikine R&D Systems, Abingdon, UK).

### PBMC qRT-PCR cytokine induction assay

For the qRT-PCR cytokine induction assay PBMC were prepared from whole blood. Heparinized (15 IU/ml) whole blood was collected under medical supervision from healthy donors after written, informed consent that complied with approved local, ethical guidelines. PBMC were separated by density gradient centrifugation in Hypaque-Ficoll (Sigma-Aldrich, Taufkirchen, Germany). Cells were washed with saline and resuspended in RPMI 1640 cell culture medium supplemented with 2 mM L-glutamine, 100 U/ml penicillin and 100 µg/ml streptomycin (Life Technologies, Darmstadt, Germany). PBMC were counted using a KX-21N blood cell counter (Sysmex, Norderstedt, Germany). PBMC were diluted to a concentration of 4 × 10^6^ PBMC/ml in RPMI culture medium. Cell suspensions (500 µl cell suspensions containing 2 × 10^6^ PBMC) were incubated in 24 well plates (BD Falcon, Heidelberg, Germany) in duplicates for 6 hours with 500 µl test solution (buffer as the negative control, challenge solution, filtrates or positive control solutions) in a humidified incubator that contained 5% CO2. After incubation, the total RNA of the PBMC was isolated using the High Pure RNA Isolation Kit (Roche Applied Science, Mannheim, Germany). Reverse transcription of the total RNA (0.5 µg) was performed using anchored oligo (dT)18 primer and the Transcriptor First Strand cDNA Synthesis Kit (Roche Applied Science). The cDNA (2 µl) was analyzed in duplicate by quantitative real-time PCR with the LightCycler 1.5 system (Roche Applied Science, Mannheim, Germany) using FastStart DNA MasterPLUS SYBR Green I (Roche Applied Science, Mannheim Germany). Single product amplification was checked by melting curve analysis supported by the LightCycler software 3.5.3. The human IL-1β primers (sense, 5′-AACAGGCTGCTCTGGGATT-3′, antisense, 5′-TCATCCTCATTGCCACTGTAA-3′) and GFAT primers (sense, 5′-TGAACGGGAAGCTCACTGG-3′, antisense, 5′-TCCACCACCCTGTTGCTGTA-3′) amplified products of 122 and 307 bp. Each PCR reaction was performed in a total volume of 20 µl (4 µl FastStart DNA MasterPLUS and primers at a concentration of 250 nM each). The instrument settings were as follows: denaturation at 95°C for 10 minutes followed by 40 cycles of denaturation at 95°C for 10 seconds, annealing at 55–60°C for 10 seconds and elongation at 72°C for 6–13 seconds (depending on the amplicon size). For absolute quantification, serial dilutions of the desired gene fragments with known copy number were performed and analyzed as external standards. Quantification was performed by online monitoring for identification of the time at which the logarithmic amplification phase was distinguishable from the background (crossing point). The crossing point signals of the unknown samples were plotted against the standard curve. The “Second derivative maximum method” supported by the LightCycler software version 3.5.3 (Roche Applied Science, Mannheim, Germany) was used for evaluation. The target gene (IL-1β) was corrected by using glyceraldehyde 3-phosphate dehydrogenase (GAPDH) as the reference gene.

### Pattern of recognition receptors bioassay

Toll-like receptors are part of the pattern of recognition receptor system, which specifically recognizes pyrogenic substances. NIH3T3 cells (mouse fibroblasts) were stably transfected with the reporter gene, secreted alkaline phosphatase (SEAP), under control of the nuclear factor (NF)κB together with specific human TLRs and their co-receptors. The TLRs used in this work included TLR1/2 and TLR2/6, which are selectively active in the presence of lipoteichoic acid and peptidoglycans, TLR4/CD14, which is selectively active in the presence of lipopolysaccharides, and TLR9, which is selectively active in the presence of bacterial DNA^13^. TLR-activity was assessed by the level of alkaline phosphatase activity induced. Alkaline phosphatase was assayed by the hydrolysis of the substrate para-nitrophenyl phosphate to the product, para-nitrophenol (which is yellow at the appropriate pH) and photometric quantification using a UV-VIS reader at 405 nm. The software SoftMaxPro (Version 5.01) was used for data recording. All PRR bioassays were conducted by contract with the Fraunhofer Institute for Interfacial Engineering and Biotechnology, Stuttgart, Germany.

### Statistics

Data are expressed as the mean ± SD. Statistical analysis was performed using an ANOVA with a Tukey's mutiple comparison post-test using GraphPad Prism 4.0. A P value of less than 0.05 was considered significant.

## Author Contributions

B.K., M.H., G.G. and R.V. designed the study. G.G. and R.S. performed the filtration assays. K.B. performed the qPCR experiments and wrote the corresponding manuscript sections. G.G., R.V., B.K. and M.H. were involved in the data interpretation. G.G., M.H., R.V. and B.K. wrote the manuscript. All authors reviewed the manuscript.
